# Favorable Cardiovascular Health Is Associated With Lower Hepatocyte Growth Factor Levels in the Multi-Ethnic Study of Atherosclerosis

**DOI:** 10.3389/fcvm.2021.760281

**Published:** 2022-01-03

**Authors:** Olatokunbo Osibogun, Oluseye Ogunmoroti, Richard A. Ferraro, Chiadi E. Ndumele, Gregory L. Burke, Nicholas B. Larson, Suzette J. Bielinski, Erin D. Michos

**Affiliations:** ^1^Department of Epidemiology, Robert Stempel College of Public Health and Social Work, Florida international University, Miami, FL, United States; ^2^Ciccarone Center for the Prevention of Cardiovascular Disease, Johns Hopkins University School of Medicine, Baltimore, MD, United States; ^3^Division of Cardiology, Johns Hopkins University School of Medicine, Baltimore, MD, United States; ^4^Division of Public Health Sciences, Wake Forest School of Medicine, Winston-Salem, NC, United States; ^5^Division of Clinical Trials and Biostatistics, Mayo Clinic College of Medicine and Science, Rochester, MN, United States; ^6^Division of Epidemiology, Mayo Clinic College of Medicine and Science, Rochester, MN, United States

**Keywords:** biomarker, cardiovascular disease, hepatocyte growth factor, ideal cardiovascular health metrics, life's simple 7, risk factors

## Abstract

**Introduction:** Hepatocyte growth factor (HGF) is a cytokine released in response to endothelial injury and a potential biomarker of cardiovascular disease (CVD) risk. We examined the association between cardiovascular health (CVH) and HGF in a multi-ethnic cohort of adults free from CVD at baseline.

**Methods:** This cross-sectional study conducted between 2020 and 2021 used MESA baseline examination data (2000–2002) from 6,490 US adults aged 45–84 years. The independent variable was CVH measured by the CVH score and number of ideal metrics. The score was derived from seven metrics: smoking, body mass index, physical activity, diet, total cholesterol, blood pressure and blood glucose. Each metric was scored 0 points (poor), 1 point (intermediate) and 2 points (ideal). The total CVH score ranged from 0 to 14. An inadequate score was 0–8, average, 9–10 and optimal, 11–14. The dependent variable was logarithmically transformed HGF. We used regression analyses to estimate associations between CVH and HGF adjusting for sociodemographic factors.

**Results:** Participants' mean (SD) age was 62 (10) years. Fifty-three percent were female. A one-unit increment in the CVH score was significantly associated with 3% lower HGF levels. Average and optimal CVH scores were significantly associated with 8% and 12% lower HGF levels, respectively, compared to inadequate scores. Additionally, a greater number of ideal metrics was associated with lower HGF levels.

**Conclusion:** Favorable CVH was significantly associated with lower HGF levels in this ethnically diverse cohort. Interventions aimed at promoting and preserving favorable CVH may reduce the risk of endothelial injury as indicated by lower serum HGF levels.

## Introduction

Hepatocyte growth factor (HGF) is a mesenchyme-derived cytokine released in response to endothelial injury and may be elevated due to insufficient compensatory mechanisms (1–4). However, HGF has cardioprotective effects in cardiac tissue through the activation of several biological pathways such as angiogenesis, anti-apoptosis, anti-inflammation and anti-fibrosis (1–4). Several studies have provided evidence that HGF is an independent predictor of coronary heart disease, heart failure, stroke and progression of atherosclerosis (5–8). Likewise, higher serum levels of HGF have been found in individuals with cardiovascular disease (CVD) risk factors e.g., smoking, obesity, hypertension and diabetes (9–13).

The American Heart Association (AHA) introduced the concept of “ideal cardiovascular health (CVH)” defined as meeting specific criteria for seven metrics. These modifiable health behaviors and factors include smoking, physical activity, body mass index (BMI), diet, blood pressure, total cholesterol and fasting plasma glucose (14, 15). The goal of the concept is to use the CVH metrics to measure and monitor CVH along with the incidence, mortality and outcomes of CVD globally (14–17). With prior studies showing that favorable CVH is associated with better endothelial function (18), lower risk of incident CVD (19) and lower levels of CVD biomarkers such as GlycA, homocysteine and cardiac troponin (20, 21), it is expected that individuals with favorable CVH would also be at lower risk of endothelial injury.

However, as little is known about the association between favorable CVH and HGF, our study aimed to analyze data from the Multi-Ethnic Study of Atherosclerosis (MESA) to examine whether participants with favorable CVH have lower levels of HGF and if the association is modified by age, sex or race/ethnicity.

## Materials and Methods

### Study Population

The methodology of the MESA study has been described elsewhere (22). In brief, the MESA study was initiated to investigate the characteristics of subclinical CVD along with the risk factors that may predict progression to clinical CVD. Between July 2000 and August 2002, MESA study personnel recruited 6,814 men and women between 45 and 84 years with no history of clinical CVD at the time of enrollment. The six recruitment centers in the U.S. were Los Angeles, CA; Chicago, IL; Baltimore, MD; St Paul, MN; Forsyth County, NC and New York, NY. The study enrolled 38% non-Hispanic White American, 12% Chinese American, 28% non-Hispanic Black American and 22% Hispanic American participants. Baseline data were collected using standardized questionnaires, physical examinations, and fasting laboratory blood tests. In this study, we included 6,490 participants from the baseline examination after the exclusion of participants with missing information for the CVH score and HGF (*n* = 324).

### Independent Variable: Cardiovascular Health

According to the AHA, a person meets the criteria for “ideal CVH” if they have ideal levels for seven modifiable health behaviors and factors which is defined as follows: (1). non-smoking; (2). BMI <25kg/m^2^; (3). weekly physical activity of 75 min of vigorous exercise or 150 min of moderate exercise; (4). a healthy diet consistent with recommended guidelines; (5). untreated total cholesterol <200 mg/dL; (6). untreated blood pressure <120/<80 mmHg and (7). untreated fasting blood glucose <100 mg/dL (14). Information on smoking status was obtained from a self-report questionnaire and defined as non-smokers (participants who had never smoked or stopped smoking >12 months), former smokers (participants who stopped smoking within the last 12 months) and current smokers. Using the measured weights and heights of participants, BMI was calculated and reported in kg/m^2^. A self-report survey instrument adapted from the Cross-Cultural Activity Participation Study (23) was used to assess physical activity. The survey contained 28 questions on time and frequency of activities during a week in the previous month. The total sum of minutes for moderate and vigorous exercise were calculated in metabolic equivalent of task (MET/min) (24). MESA study personnel used a 120-item validated food frequency questionnaire adapted from the Insulin Resistance Atherosclerosis Study instrument (25, 26) to collect data on dietary habits. Based on recommended dietary guidelines, a healthy diet comprised of fruits and vegetables, fish, whole grains, intake of sodium <1,500 mg/day and sugar-sweetened beverages ≤450 kcal (36 oz.)/week 14. Fasting blood samples were collected after a 12-h fast to measure total cholesterol (mg/dL) and blood glucose (mg/dL) levels. MESA study personnel used the cholesterol oxidase method to measure total cholesterol in ethylenediaminetetraacetic (EDTA) plasma using a centrifugal analyzer (Roche). While blood glucose was measured by the glucose oxidase method on a Vitros analyzer (Johnson & Johnson). Three blood pressure readings were taken from participants after 5 minutes was of rest in a seated position using a Dinamap model Pro 100 automated oscillometric sphygmomanometer (Critikon) and the mean of the last two blood pressure readings was used in the analysis.

### Dependent Variable: Hepatocyte Growth Factor

MESA study personnel obtained peripheral blood samples from participants for the measurement of HGF at baseline. The samples were stored at −70°C in serum separated by centrifugation within 30 minutes of collection (8). Serum HGF was measured by quantitative sandwich enzyme-linked immunosorbent assay using the Human soluble HGF/CD62P Immunoassay kit (R&D Systems, Minneapolis, MN) with a lower limit of detection of 40 pg/mL (5, 8). For lyophilized manufacturer's controls, the inter-assay laboratory coefficients of variation (CV) were 12.0, 8.0 and 7.4% at mean concentrations of 687, 2,039 and 4,080 pg/mL, respectively (5, 8). For in-house pooled serum control, the inter-assay laboratory CV was 10.4% at a mean concentration of 688 pg/mL (5, 8). HGF was logarithmically transformed for this analysis because of the skewness of the data.

### Covariates

Baseline sociodemographic factors included as covariates were age, sex, race/ethnicity, education, income, health insurance status and study site. Age was assessed as a continuous variable while sex was categorized into male and female. Study participants self-classified themselves as either non-Hispanic White, Chinese, non-Hispanic Black, or Hispanic American. Education and income were grouped into 9 and 13 categories, respectively for analysis but both variables were dichotomized in the descriptive statistics reported in Table 1. We categorized health insurance status as participants with or without health insurance.

**Table 1:** Characteristics of study participants at the MESA baseline exam (2000–2002) by cardiovascular health score.

	**Total**	**Inadequate**	**Average**	**Optimal**	***P*-value**
	***N* = 6,490**	***n* = 3,069**	***n* = 2,115**	***n* = 1,306**	
**HGF**, pg/mL	903 (755–1,085)	969 (821–1,159)	870 (740–1,036)	807 (678–962)	–
**Age**, years	62 (10)	63 (10)	62 (11)	60 (10)	
<65 years	3,703 (57%)	1,682 (55%)	1,202 (57%)	819 (63%)	<0.001
≥65 years	2,787 (43%)	1,387 (45%)	913 (43%)	487 (37%)	
**Sex**					
Male	3,067 (47%)	1,460 (48%)	1,000 (47%)	607 (46%)	0.80
Female	3,423 (53%)	1,609 (52%)	1,115 (53%)	699 (54%)	
**Race/ethnicity**					
Non-Hispanic White	2,534 (39%)	977 (32%)	905 (43%)	652 (50%)	<0.001
Chinese-American	796 (12%)	216 (7%)	319 (15%)	261 (20%)	
Non-Hispanic Black	1,706 (26%)	1,035 (34%)	472 (22%)	199 (15%)	
Hispanic	1,454 (22%)	841 (27%)	419 (20%)	194 (15%)	
**Education**					
≥ Bachelor's degree	2,329 (36%)	795 (26%)	833 (39%)	701 (54%)	<0.001
< Bachelor's degree	4,161 (64%)	2,274 (74%)	1,282 (61%)	605 (46%)	
**Income**					
≥$40,000	3,206 (49%)	1,267 (41%)	1,122 (53%)	817 (63%)	<0.001
< $40,000	3,284 (51%)	1,802 (59%)	993 (47%)	489 (37%)	
**Health insurance**					
Yes	5,909 (91%)	2,781 (91%)	1,940 (92%)	1,188 (91%)	0.39
No	581 (9%)	288 (9%)	175 (8%)	118 (9%)	

*HGF, hepatocyte growth factor; MESA, multi-ethnic study of atherosclerosis. Data were presented as mean (SD), median (IQR) or n (%). Percentages were rounded up to whole numbers. P-values indicate differences across the CVH score categories*.

### Statistical Analysis

All analyses were conducted with version 15.0 of STATA (StataCorp LP, College Station, TX) between 2020 and 2021 and a two-sided *p* value < 0.05 was considered statistically significant. We reported the characteristics of participants for the total study population and by the CVH score. In Supplementary Material, we also presented the baseline characteristics of study participants by HGF tertiles. For categorical variables, we reported frequencies with percentages while for continuous variables we reported medians with interquartile range (IQR) or means with standard deviation (SD). We used the chi-square and ANOVA tests to compare baseline characteristics by the CVH score for categorical and continuous variables, respectively. The individual CVH metrics were categorized into poor, intermediate and ideal as shown in the Supplementary Table S1 (14). We assigned points to the categories as follows: 0 points for poor, 1 point for intermediate and 2 points for ideal with a total CVH score of 0 to 14 (27). The CVH score was further categorized as inadequate (0–8), average (9–10) and optimal (11–14) based on prior studies (28, 29).

We estimated the associations between CVH and log (HGF) using multiple linear regression. We fitted two separate models. Model 1 was unadjusted while model 2 was adjusted for sociodemographic factors [age, sex, race/ethnicity, education (9 categories), income (13 categories), health insurance status and study site]. We analyzed CVH using three measures: the continuous CVH score, the categorical CVH score and the number of ideal metrics. Of note, the number of ideal metrics was derived by counting the number of individual CVH metrics in the ideal category. We reported the percentage difference of the geometric mean of log (HGF) and the corresponding 95% confidence intervals (CI) for each measure of CVH. The percentage difference was calculated from the exponentiated beta coefficient minus 1 multiplied by 100 ([Exp (β) – 1]^*^100). For the CVH score and the number of ideal metrics, the reference groups were the “inadequate score” and “zero ideal metrics,” respectively. We assessed effect modification by age (<65 vs. ≥65 years), sex and race/ethnicity by inserting interaction terms in model 2. Furthermore, we examined the associations between the individual CVH metrics and log (HGF) using multiple linear regression. The models were adjusted as previously described. We reported the percentage difference of the geometric mean of log (HGF) and the corresponding 95% CI for the intermediate and ideal categories of each CVH metric compared to the poor category.

## Results

**Figure 1:**
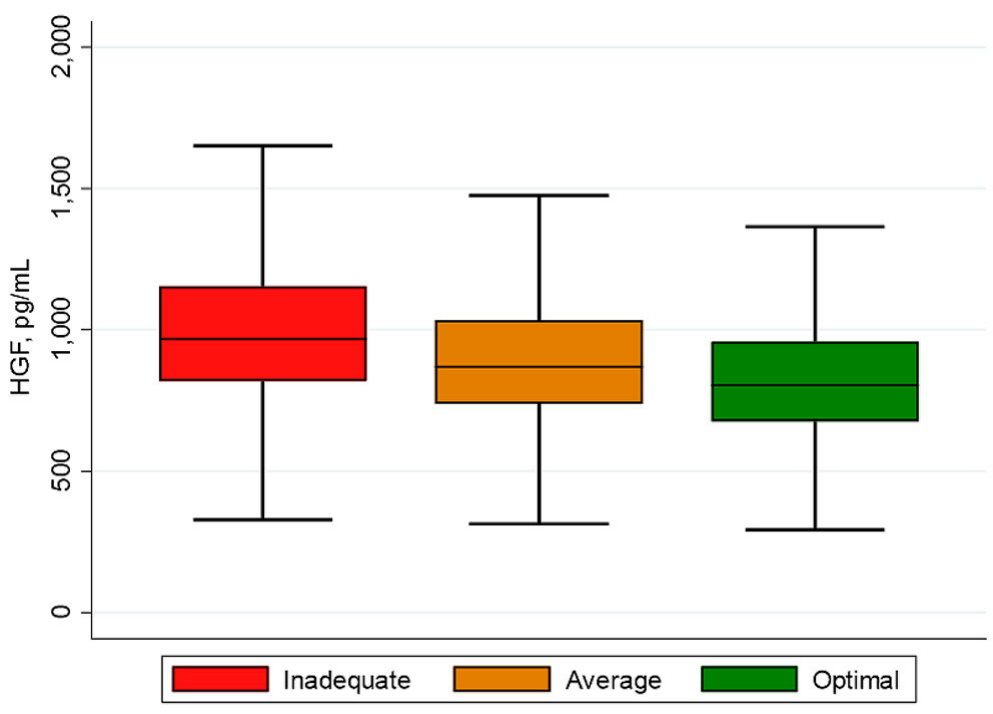
Box plot of HGF by CVH scores. The lower and upper boundaries of the rectangles denote the 25th and 75th percentiles while the horizontal line within the rectangles is the median. Lines extend from the rectangles to the smallest and largest values within 1.5 × interquartile range.

Most of the baseline characteristics of study participants (*N* = 6,490) varied by the categories of the CVH score and HGF tertiles as reported in Table 1 and in the Supplementary Table S2. The mean age (SD) of participants was 62 (10) years and 53% were female. As illustrated in Figure 1, participants with inadequate CVH scores had higher HGF levels compared to those with optimal CVH scores. Additionally, the mean (SD) of CVH scores declined across tertiles of HGF [9.4 (2.0), 8.6 (2.1) and 7.8 (2.2) for tertiles 1–3, respectively] as shown in the Supplementary Table S2.

Table 2 shows the associations between CVH and HGF. A one-unit increment in the continuous measure of the CVH score was significantly associated with 3% lower HGF levels after adjusting for sociodemographic factors. For the categorical measure of the CVH score, the average and optimal scores were significantly associated with 8% and 12% lower HGF levels, respectively, compared to inadequate scores. In addition, a greater number of ideal metrics was associated with lower HGF levels. For example, study participants with 5 and 6–7 ideal metrics had 18% and 23% lower HGF levels, respectively. These associations were slightly attenuated when compared to the unadjusted model. The tests for effect modification by age, sex and race/ethnicity were not statistically significant (*p* > 0.05).

**Table 2:** Associations between cardiovascular health and hepatocyte growth factor in MESA (2000–2002), *N* = 6,490.

	**Model 1**	**Model 2**
	**% difference (95% CI)**	**% difference (95% CI)**
CVH score, continuous	**−4 (−4**, −3)*	**−3 (−3**, −3)*
CVH score, categorical		
Inadequate	Reference	Reference
Average	**−10 (−11**, −9)*	**−8 (−9**, −6)*
Optimal	**−17 (−18**, −16)*	**−12 (−14**, −11)*
Number of ideal metrics		
0	Reference	Reference
1	**–**3 (**–**12, 7)	**–**4 (**–**13, **–**5)
2	**–**6 (**–**15, 3)	**–**6 (**–**14, 3)
3	**−13 (−21**, −5)**	**−12 (−20**, −4)**
4	**−18 (−25**, −10)*	**−15 (−23**, −7)*
5	**−22 (−29**, −15)*	**−18 (−25**, −10)*
6–7	**−29 (−36**, −22)*	**−23 (−30**, −16)*

*CI, confidence interval; CVH, cardiovascular health; MESA, multi-ethnic study of atherosclerosis*.

*CVH score ranges from 0 to 14. Inadequate score, 0–8; average, 9–10; optimal, 11–14*.

% difference is the % difference of the geometric mean of HGF calculated from [Exp (β) – 1]*100.

Statistically significant results are in bold font: *P < 0.001; **P < 0.01.

*Model 1: Unadjusted*.

*Model 2: Adjusted for age, sex, race/ethnicity, education, income, health insurance and study site*.

*Interpretation: A one-unit increment in the CVH score was significantly associated with 3% lower HGF levels in model 2 [−3 (−3, −3)]. No significant interaction by age, sex or race/ethnicity*.

Table 3 shows the associations between the individual CVH metrics and HGF. Participants in the ideal category for smoking had 11% lower HGF levels in the adjusted model compared to those in the poor category of smoking which was a stronger association in comparison to the unadjusted model. Although slightly attenuated, ideal BMI and physical activity were associated with an adjusted 12% and 4% lower HGF levels, respectively. Participants who reported eating an ideal diet had 7% lower HGF levels with no appreciable change from the unadjusted model. However, those who met the ideal criteria for total cholesterol had an adjusted 3% higher HGF levels. Ideal blood pressure and blood glucose were associated with 7% and 11% lower HGF levels, respectively, although the estimates were slightly attenuated in comparison to the estimates from the unadjusted model.

**Table 3:** Associations between the cardiovascular health metrics and hepatocyte growth factor in MESA (2000–2002), *N* = 6,490.

	**Model 1**		**Model 2**	
	**% difference (95% CI)**		**% difference (95% CI)**	
	**Intermediate vs Poor**	**Ideal vs Poor**	**Intermediate vs Poor**	**Ideal vs Poor**
Smoking	–**10 (**–**15**, –4)^**^	–**9 (**–**11**, –7)^*^	–**11 (**–**16**, –5)^*^	–**11 (**–**12**, –9)^*^
Body mass index	–**9 (**–**10**, –7)^*^	–**14 (**–**16**, –13)^*^	–**8 (**–**10**, –7)^*^	–**12 (**–**14**, –11)^*^
Physical activity	–**3 (**–**5**, –1)^**^	–**7 (**–**8**, –5)^*^	−1 (−3, 1)	–**4 (**–**6**, –3)^*^
Diet	–**2 (**–**3**, –1)^**^	–**7 (**–**13**, –1)^**^	–**2 (**–**3**, –1)^**^	–**7 (**–**13**, –1)^**^
Total cholesterol	1 (−2, 3)	0 (−3, 2)	2 (0.1, 4)^**^	3 (1, 5)^**^
Blood pressure	–**5 (**–**7**, –3)^*^	–**12 (**–**13**, –11)^*^	–**2 (**–**4**, –1)^**^	–**7 (**–**9**, –6)^*^
Blood glucose	–**5 (**–**7**, –2)^*^	–**15 (**–**17**, –13)^*^	–**3 (**–**5**, –1)^**^	–**11 (**–**13**, –9)^*^

*CI, confidence interval; MESA, multi-ethnic study of atherosclerosis*.

% difference is the % difference of the geometric mean of HGF calculated from [Exp (β) – 1]*100.

*Statistically significant results are in bold font:*

* *P < 0.001;*

** *P < 0.01*.

*Model 1: unadjusted*.

*Model 2: adjusted for age, sex, race/ethnicity, education, income, health insurance and study site*.

*Interpretation: Ideal smoking was significantly associated with 11% lower HGF levels in model 2 [−11 (−12, −9)]*.

## Discussion

In this cross-sectional analysis of 6,490 adults free of clinical CVD at baseline, we found that participants with higher CVH scores or greater number of ideal metrics had lower levels of HGF. Similarly, we found that participants who met the ideal criteria for the individual CVH metrics had lower levels of HGF except for total cholesterol where ideal levels were associated with higher levels of HGF.

Prior studies in the MESA cohort have examined the association between HGF and CVD. One of these studies found that the risk of incident stroke was 17% greater with every standard deviation increase in circulating HGF independent of sociodemographic and CVD risk factors (7). Another study from the same cohort found that every standard deviation increase in circulating HGF was associated with 12% greater odds of having any coronary artery calcium and 20% increased risk of coronary heart disease after adjusting for traditional CVD risk factors (30). Furthermore, in another analysis from the MESA cohort, the risk of heart failure and heart failure with preserved ejection fraction was 59% and 90% higher, respectively, for participants with HGF levels in the 3rd tertile compared to those in the 1st tertile, independent of sociodemographic and CVD risk factors (31). These studies support our findings of an association between favorable CVH and lower levels of HGF since HGF is elevated in CVD as a response to endothelial damage (7). Factors that lead to HGF release may mediate the association between poorer CVH and incident CVD events.

In addition, our results showing that ideal CVH metrics (excluding total cholesterol) were associated with lower levels of HGF are supported by the findings from prior research. For example, Chen et al. (32) demonstrated that a history of current or previous smoking increases HGF expression in non-tumor lung tissue. The authors postulated that smoking increases HGF levels by activating HGF gene expression, however, the mechanism by which this process occurs is yet to be determined (32). Rehman et al. (10) showed that obesity was associated with more than three times increase in HGF levels and this finding was correlated linearly with BMI. The presence of excess adipose tissue may explain the association between poor BMI and elevated HGF (10). The explanation for the association between ideal physical activity and lower HGF levels is still under investigation.

In contrast to the inverse association for the other CVH metrics, we found that ideal cholesterol was paradoxically associated with higher HGF. This finding has also been observed in another study by Hiratsuka et al. (13) where participants with higher HGF levels in the fourth quartile had lower total cholesterol levels compared to those in the first quartile. However, the biological pathway responsible for the association between higher HGF levels and lower cholesterol levels is still unknown. Nakamura et al. (11) reported HGF levels in participants with hypertension was significantly higher than in participants without hypertension. In addition, Bancks et al. (12) observed that higher levels of circulating HGF was significantly associated with incident type 2 diabetes in the MESA cohort. These findings are not surprising given that hypertension and diabetes are associated with endothelial damage and dysfunction which may lead to elevated HGF levels (11, 12).

Our study revealed that poorer CVH was associated with higher HGF levels, therefore measuring HGF levels may be of utility in identifying people in poorer CVH because prior research has provided insight into the clinical significance of HGF as a risk marker for subclinical and clinical CVD (7, 8, 30). With the increasing burden and cost of managing CVD, effective interventions that promote favorable CVH are needed (33). These public health intervention programs should encourage lifestyle modification which will reduce the risk of developing CVD through several potential mechanisms including the reduction of endothelial injury as indicated by lower HGF levels (33).

### Strengths and Limitations

Although this study was conducted in a large multi-ethnic population using standardized methods for data collection, our study findings should be interpreted with the consideration of some limitations. First, because this study is cross-sectional, we cannot determine temporality or make causal inferences for the association between CVH and HGF. Second, information collected from study participants for the individual CVH metrics such as smoking, diet and physical activity was from self-report questionnaires and may be subject to recall bias. Third, MESA study personnel assessed circulating HGF and not tissue-specific HGF, however, prior studies have shown that both measurements are correlated (8, 34). Fourth, selection of our study population was non-randomized. In addition, the participants were free of CVD at the time of study enrollment and may have been healthier compared to the general population, therefore limiting the generalizability of our findings. Finally, CVH was measured only at baseline and may not reflect the future CVH status of study participants.

## Conclusion

In this multicenter cross-sectional study of adults free of CVD at baseline, we found that favorable CVH was associated with lower levels of HGF. Interventions aimed at promoting and preserving favorable CVH may reduce endothelial injury and decrease CVD risk as indicated by lower serum HGF levels. However, there is need for prospective studies to examine the relationship between CVH and HGF to determine whether HGF levels can be modified by improvements in the CVH metrics. More research is needed to provide further understanding of the underlying mechanisms driving the associations.

## Data Availability Statement

The datasets presented in this article are not readily available because there are restrictions on sharing the actual MESA database because it has patient identifiers and protected health information and thus is subject to IRB approval. We have signed a data use agreement (DUA) with the MESA Coordinating Center that we cannot directly share data and that anyone with access to the data has to follow MESA policies and procedures for data use. We are not allowed to personally share the datasets and have to keep them stored securely in a Cloud using encrypted software for protection. However, other researchers may obtain the minimal dataset required to replicate our study's findings by submitting a proposal through the NIH BioLINCC (https://biolincc.nhlbi.nih.gov/studies/mesa/). Additionally, one can apply to the MESA Coordinating Center to become a new investigator after signing a DUA. The findings of this study should be easily reproducible through the methods described in this paper. Requests to access the datasets should be directed to Craig Johnson (wcraigj@uw.edu) at the Coordinating Center or at https://www.mesa-nhlbi.org/.

## Ethics Statement

The MESA study protocol was approved by the Institutional Review Boards (IRB) at each recruitment center and informed consent was given by all participants. At Johns Hopkins University, this study was approved by the Johns Hopkins School of Medicine IRB applicant number NA_00030361. The participants provided their written informed consent to participate in this study.

## Author Contributions

OOs, OOg, and EM: study conception and design and drafting of manuscript. OOs and OOg: acquisition of data and analysis. OOs, OOg, RF, CN, GB, NL, SB, and EM: interpretation of data and critical revision and approval of final version submitted. All authors contributed to the article and approved the submitted version.

## Funding

The MESA study was supported by contracts HHSN268201500003I, N01-HC-95159, N01-HC-95160, N01-HC-95161, N01-HC-95162, N01-HC-95163, N01-HC-95164, N01-HC-95165, N01-HC-95166, N01-HC-95167, N01-HC-95168, and N01-HC-95169 from the National Heart, Lung and Blood Institute (NHLBI) and by grants UL1-TR-000040, UL1-TR-001079, and UL1-TR-001420 from the National Center for Advancing Translational Sciences. HGF measurement was funded by R01HL98077. EM was additionally funded by the Amato Fund for Women's Cardiovascular Health at Johns Hopkins University. The funding sources had no role in the study design; collection, analysis and interpretation of data; writing the report; and the decision to submit the report for publication. The specific roles of the authors are articulated in the author contributions section.

## Conflict of Interest

The authors declare that the research was conducted in the absence of any commercial or financial relationships that could be construed as a potential conflict of interest.

## Publisher's Note

All claims expressed in this article are solely those of the authors and do not necessarily represent those of their affiliated organizations, or those of the publisher, the editors and the reviewers. Any product that may be evaluated in this article, or claim that may be made by its manufacturer, is not guaranteed or endorsed by the publisher.
