# Supplementary material for: Favorable Cardiovascular Health Is Associated With Lower Hepatocyte Growth Factor Levels in the Multi-Ethnic Study of Atherosclerosis
Source: Front Cardiovasc Med. 2022 Jan 3;8:760281. doi: 10.3389/fcvm.2021.760281 (PMC8761906; doi:10.3389/fcvm.2021.760281)
Supplement: Supplementary file 1 [file Data_Sheet_1.docx]

Supplementary Material

Table S1: Distribution of the cardiovascular health metrics.

Table S2: Characteristics of study participants at the MESA baseline exam (2000-2002) by HGF tertiles.

|  | **Table S1. Distribution of the CVH metrics** | |  |
| --- | --- | --- | --- |
| **CVH metrics** | **Point** | **Definition** | **% MESA Participants N = 6,490** |
| **Smoking** | 0 | Current smoker | 837 (13%) |
|  | 1 | Former smoker, quit ≤12 months ago | 80 (1%) |
|  | 2 | Never smoker or quit >12 months ago | 5,573 (86%) |
| **Body Mass Index** | 0 | ≥30 kg/m^2^ | 2,066 (32%) |
|  | 1 | 25.0–29.99 kg/m^2^ | 2,552 (39%) |
|  | 2 | <25.0 kg/m^2^ | 1,872 (29%) |
| **Physical Activity** | 0 | No exercise | 1,480 (23%) |
|  | 1 | 1–149 min of moderate exercise or 1–74 min of vigorous exercise/week | 1,124 (17%) |
|  | 2 | 150+ min of moderate exercise or 75+ min of vigorous exercise/week | 3,886 (60%) |
| **Diet** | 0 | 0–1 components of healthy diet | 2,934 (45%) |
|  | 1 | 2–3 components of healthy diet | 3,486 (54%) |
|  | 2 | 4–5 components of healthy diet | 70 (1%) |
| **Total Cholesterol** | 0 | ≥240 mg/dL | 870 (13%) |
|  | 1 | 200–239 mg/dL or treated to <200mg/dL | 2,533 (39%) |
|  | 2 | <200 mg/dL, unmedicated | 3,087 (48%) |
| **Blood Pressure** | 0 | SBP ≥140 mmHg or DBP ≥90 mmHg | 2,431 (37%) |
|  | 1 | SBP 120–139 mmHg or DBP 80–89 mmHg or treated to <120/80 mm Hg | 1,816 (28%) |
|  | 2 | <120/80 mm Hg, unmedicated | 2,243 (35%) |
| **Blood Glucose** | 0 | ≥126 mg/dL fasting | 696 (11%) |
|  | 1 | 100–125 mg/dL fasting or treated to <100 mg/dL | 986 (15%) |
|  | 2 | <100 mg/dL fasting, unmedicated | 4,808 (74%) |
| Adapted from Lloyd Jones et al 2010 and Unger et al 2014.  Abbreviations: CVH, cardiovascular health; DBP, diastolic blood pressure, MESA, Multi-Ethnic Study of Atherosclerosis and SBP, systolic blood pressure. Poor=0 points; Intermediate=1 point; ideal =2 points. *When combining vigorous and moderate exercise, vigorous exercise was weighted double**.** | | | |

| **Table S2. Characteristics of study participants at the MESA baseline exam (2000-2002) by HGF tertiles.** | | | | | |
| --- | --- | --- | --- | --- | --- |
|  | Total | 1^st^ Tertile  \ | 2^nd^ Tertile | 3^rd^ Tertile | P-value |
|  | N = 6,490 | n = 2,164 | n = 2,163 | n = 2,163 |  |
| HGF, pg/mL | 903  (755-1,085) | 699  (623-755) | 903  (855-956) | 1,170 (1,085-1,325) | - |
| Age, years | 62 (10) | 59 (9) | 62 (10) | 65 (10) | < 0.001 |
| < 65 years | 3,703 (57%) | 1,504 (70%) | 1,235 (57%) | 964 (45%) | < 0.001 |
| ≥ 65 years | 2,787 (43%) | 660 (31%) | 928 (43%) | 1,199 (55%) |  |
| Sex |  |  |  |  |  |
| Male | 3,067 (47%) | 1,111 (51%) | 989 (46%) | 967 (45%) | < 0.001 |
| Female | 3,423 (53%) | 1,053 (49%) | 1,174 (54%) | 1,196 (55%) |  |
| Race/Ethnicity |  |  |  |  |  |
| Non-Hispanic White | 2,534 (39%) | 946 (44%) | 800 (37%) | 788 (36%) |  |
| Chinese-American | 796 (12%) | 398 (18%) | 257 (12%) | 141 (7%) | < 0.001 |
| Non-Hispanic Black | 1,706 (26%) | 560 (26%) | 605 (28%) | 541 (25%) |  |
| Hispanic | 1,454 (22%) | 260 (12%) | 501 (23%) | 693 (32%) |  |
| Education |  |  |  |  |  |
| ≥ Bachelor's degree | 2,329 (36%) | 1,012 (47%) | 780 (36%) | 537 (25%) | < 0.001 |
| < Bachelor’s degree | 4,161 (64%) | 1,152 (53%) | 1,383 (64%) | 1,626 (75%) |  |
| Income |  |  |  |  |  |
| ≥$40,000 | 3,206 (49%) | 1,305 (60%) | 1,051 (49%) | 850 (39%) | < 0.001 |
| <$40,000 | 3,284 (51%) | 859 (40%) | 1,112 (51%) | 1,313 (61%) |  |
| Health insurance |  |  |  |  |  |
| Yes | 5,909 (91%) | 1,968 (91%) | 1,965 (91%) | 1,976 (91%) | 0.82 |
| No | 581 (9%) | 196 (9%) | 198 (9%) | 187 (9%) |  |
| CVH score, continuous | 8.6 (2.2) | 9.4 (2.0) | 8.6 (2.1) | 7.8 (2.2) | < 0.001 |
| CVH score, categorical |  |  |  |  |  |
| Inadequate | 3,069 (47%) | 707 (33%) | 1,029 (48%) | 1,333 (62%) |  |
| Average | 2,115 (33%) | 803 (37%) | 721 (33%) | 591 (27%) | < 0.001 |
| Optimal | 1,306 (20%) | 654 (30%) | 413 (19%) | 239 (11%) |  |
| Abbreviation: CVH, Cardiovascular Health; HGF, Hepatocyte Growth Factor; MESA, Multi-Ethnic Study of Atherosclerosis. Data were presented as mean (SD), median (IQR) or n (%). Percentages were rounded up to whole numbers. P values indicate differences across the CVH score categories. | | | | | |
